# Genetics Reveal the Origin and Timing of a Cryptic Insular Introduction of Muskrats in North America

**DOI:** 10.1371/journal.pone.0111856

**Published:** 2014-10-31

**Authors:** Alexis M. Mychajliw, Richard G. Harrison

**Affiliations:** 1 Department of Biology, Stanford University, Stanford, California, United States of America; 2 Department of Ecology & Evolutionary Biology, Cornell University, Ithaca, New York, United States of America; University of Illinois at Urbana-Champaign, United States of America

## Abstract

The muskrat, *Ondatra zibethicus*, is a semiaquatic rodent native to North America that has become a highly successful invader across Europe, Asia, and South America. It can inflict ecological and economic damage on wetland systems outside of its native range. Anecdotal evidence suggests that, in the early 1900s, a population of muskrats was introduced to the Isles of Shoals archipelago, located within the Gulf of Maine, for the purposes of fur harvest. However, because muskrats are native to the northeastern coast of North America, their presence on the Isles of Shoals could be interpreted as part of the native range of the species, potentially obscuring management planning and biogeographic inferences. To investigate their introduced status and identify a historic source population, muskrats from Appledore Island of the Isles of Shoals, and from the adjacent mainland of Maine and New Hampshire, were compared for mitochondrial cytochrome *b* sequences and allele frequencies at eight microsatellite loci. Appledore Island muskrats consistently exhibited reduced genetic diversity compared with mainland populations, and displayed signatures of a historic bottleneck. The distribution of mitochondrial haplotypes is suggestive of a New Hampshire source population. The data presented here are consistent with a human-mediated introduction that took place in the early 1900s. This scenario is further supported by the zooarchaeological record and island biogeographic patterns. This is the first genetic study of an introduced muskrat population within US borders and of any island muskrat population, and provides an important contrast with other studies of introduced muskrat populations worldwide.

## Introduction

Questions regarding the origin, distribution, and maintenance of insular fauna have been central to the study of evolutionary biology since the time of Charles Darwin and Alfred Russell Wallace. Island systems are frequently touted as natural laboratories for the study of fundamental ecological and evolutionary processes [1]. However, attempts to delimit the biodiversity of island systems can be confounded by “cryptic” invasive species, i.e., a human-aided, otherwise nonnative species may be mistaken for a native island species [2]. This has the effect of inflating the number of endemic taxa recorded and can mislead biogeographic inferences (e.g., by distorting species-area curves) or conservation priorities. Because invasive species are a major cause of extinction in endemic insular fauna [3]–[4], determining whether a species is native or introduced to an island ecosystem is critical to informing its subsequent protection or eradication [5]–[6].

The colonization of islands, with or without human mediation, generally involves only a small subsample of the mainland source population. Information concerning initial founding conditions is typically unknown and traditional methods, such as the use of historical or zooarchaeological records, often fail to provide comprehensive resolution. Because morphological evidence of endemism can be ambiguous [2], [6], the combined use of microsatellite and mitochondrial DNA analyses has become a powerful approach for identifying past bottlenecks, source populations, and evaluating the native status of a potentially cryptically introduced species [e.g., 5].

**Figure 1:**
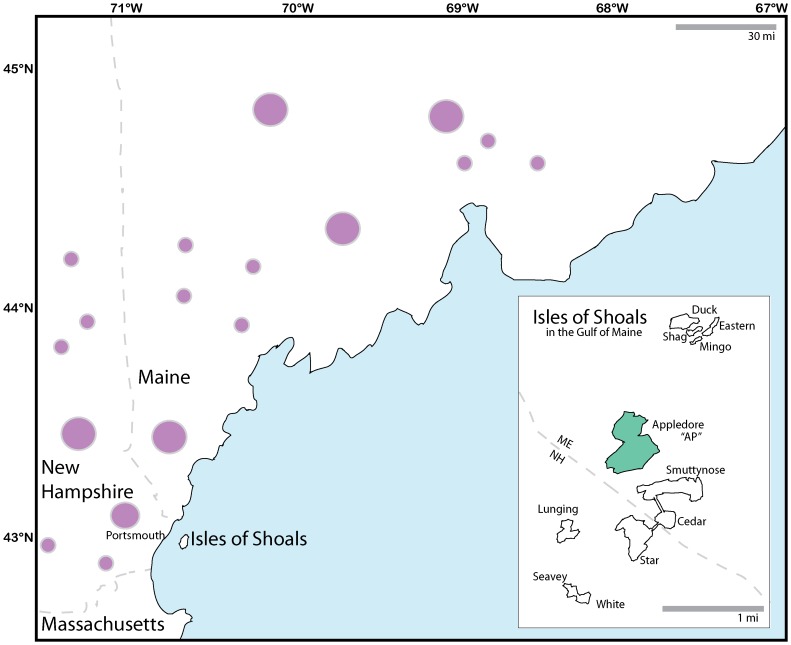
Map of study region depicting Appledore Island within the Isles of Shoals and in relation to the northeastern US coastline. Dashed gray lines represent state boundaries. Circles represent generalized population sampling locations on the mainland, with larger circles indicative of 4+ samples, and smaller circles of one. Appledore Island is highlighted in green (see Figure S1 for fine-scale Appledore Island sampling).

The population of muskrats, *Ondatra zibethicus* inhabiting the Isles of Shoals archipelago in the Gulf of Maine (Figure 1) likely constitutes such a cryptic introduction. Like many furbearing mammals, muskrats have often been transported by humans for fur farming, and are now invasive across Europe, Russia, China, Argentina, and Chile [7]. Although their native range encompasses most of North America, the fur trade has produced human-mediated translocations even within the US, and these have been documented in the historical record in California, Louisiana, Alaska, and New York [7]. Anecdotes from fur trappers and government officials suggest that muskrats have been present on the Isles of Shoals since the 1930s, and they appear only recently in the zooarchaeological record, as compared with Norway rats that have an older record paralleling the initial use of the Isles by early settlers in the 1600s (N. Hamilton *personal communication*, [8]–[9]).

Despite the ubiquity of muskrats as a globally invasive species and the economic importance of muskrat fur for many rural communities [10], there is a paucity of genetic information available regarding this species in both their native and non-native range. Laurence et al. [11] have described basic genetic variability in North America, which serves as the only baseline for comparison with worldwide invasive populations. As muskrats are known to negatively impact the ecosystems they invade by causing damage to irrigation structures, predating fish fry, destroying agricultural crops, and outcompeting native small mammals (such as desmans in Russia [7]), it is critical to build a more comprehensive dataset of the genetic composition of muskrat populations for comparative applications.

This study seeks to illuminate the origins of muskrats on the Isles of Shoals archipelago from potential mainland source populations. The Isles are already known to harbor numerous non-native species: for example, 42% of plant species are non-native, and 23 of these are classified as invasive in New England [12]. Within the northern Gulf of Maine, there are several confirmed cases of intentional introductions of game and fur-bearing species, including deer, beavers, and rabbits, made in the first quarter of the century on large coastal islands [13]. However, the geologic history of the archipelago, the native range of muskrats along the coastline and on large coastal islands in the northern Gulf of Maine, and the strong swimming abilities of muskrats have contributed to their cryptic status. Muskrats are the only species of nonvolant mammal (aside from the unmistakably invasive Norway rat) established on the archipelago [14].

The archipelago is located 11 km off the northeastern United States coastline and is bisected by the border between Maine (ME) and New Hampshire (NH) (Figure 1). During a sea level minimum approximately 11,000 years before present (YBP), a land bridge connected the islands to the mainland; the islands were then isolated as sea levels rose to modern levels 7,000 YBP [15]. The Isles were heavily used by humans in the 17^th^ century for cod fishing and supported a year-round human population of 600–1200 people [12]. The three largest islands—Appledore (AP; 40 ha), Smuttynose (10 ha), and Star Island (19 ha)—continue to sustain seasonal human activities on a much smaller scale [12].

This study contrasts two hypotheses of natural colonization that would produce distinctive genetic patterns (colonization via land bridge 11,000–7,000 YBP, recurrent colonizations from migration by swimming) with the anecdotally informed but otherwise untested hypothesis of a human-mediated muskrat introduction in the early 1900s. It aims to illustrate the usefulness of a combined mitochondrial and microsatellite DNA approach and provide a framework of multiple complementary statistics for the detection of a cryptically introduced population. Through this approach, we compare expectations under each hypothesis, including timing of bottleneck, endemism of haplotypes, degree of gene flow, and levels of genetic diversity and differentiation, to distinguish the most likely scenario of muskrat colonization. For example, if muskrats colonized naturally via a land bridge, there would be no signatures of a recent bottleneck, very high levels of genetic differentiation with the mainland, and we would likely find at least one mitochondrial haplotype endemic to AP due to an early divergence followed by isolation over several thousand years. Conversely, recurring natural colonization events would be reflected in low genetic differentiation and a high maintenance of genetic diversity due to continual gene flow. A human-mediated colonization in the 1900s would display different signatures than either natural colonization mechanism, primarily differing in the timing of the population bottleneck, absence of an endemic haplotype, and magnitude of gene flow and differentiation with the mainland.

## Materials and Methods

### Ethics Statement

This study was approved by the Cornell University Institutional Animal Care & Use Committee, and operated under IACUC General Operation of the Mammalogy Collection at Cornell University protocol #2006-0156. Samples of AP muskrats were collected on private property owned by the Shoals Marine Laboratory of Cornell University, under Maine Department of Inland Fisheries and Wildlife, Wildlife Scientific Collection permit #2011-349. All AP samples were collected under strict adherence to American Society of Mammalogy guidelines [16]. Muskrats are not endangered, and they are regularly harvested for fur in the states of ME and NH. All sampled individuals on AP were subsequently released. ME and NH samples were donated by fur-trappers acting under valid state collection permits.

### Sample Collection

Muskrats were non-lethally sampled via tail clips (<1 cm) on Appledore Island (AP), Isles of Shoals, ME, from June to July 2011 using Havahart live-traps (Figure S1; Table S1). Muskrat houses, burrows, and latrines were surveyed prior to sampling to ensure appropriate geographic coverage and to avoid sampling of parent-offspring within a territory. Mark-recapture studies conducted in 1984 [17] demonstrated similar muskrat habitat use across the island and suggested a population density of 27±5.5 muskrats/ha, yielding a tentative population size estimate of 860–1,300 individuals. A total of 43 AP samples were collected and stored in absolute ethanol.

Members of state trapping associations donated tissue samples (stored in absolute ethanol) from the mainland (46 from ME and 18 from NH; Figure 1; Table S1). Our sample sizes are consistent with those used in a study of muskrat microsatellite diversity across states of the United States (e.g., 36 samples from New York) and provinces of Canada (e.g., 20 samples from Alberta) [11].

### DNA Extraction and Amplification

A DNeasy Blood & Tissue Kit (Qiagen) was used to extract DNA from a total of 107 tissue samples. Mitochondrial DNA sequences of the cytochrome *b* gene were generated for 79 individuals: 33 from AP, 31 from ME and 15 from NH. Primers were designed using Primer Select (DNASTAR) and a pre-existing cytochrome *b* gene sequence, Genbank Accession #AF119277 [18]. PCR amplifications were performed in 10 µL reactions containing 1 µL template DNA, 1 U of platinum *Taq* polymerase, 1 µL PCR buffer, 2 mM MgCl_2_, 0.2 µM of each primer (*OzbFW* 5′CACTCATTCATCGACCTCCCAAC3′; *OzbREV* 5′TGGGTATGAAGATAATGATAATGGCAAAGTA3′) and 0.2 mM dNTP. PCR reactions included an initial denaturation at 94°C for 5 minutes, followed by 35 cycles of 94°C for 15 seconds, 50°C for 15 seconds, and 72°C for 1 minute, with a final extension of 72°C for 5 minutes. PCR products were cleaned using ExoSAP-IT and CleanSEQ (Agencourt), and amplified using a Big Dye Cycle Sequencing kit (Applied Biosystems). Sequencing was performed using an Applied Biosystems 3730 DNA Analyzer at the Life Sciences Core Laboratories Center at Cornell University (http://cores.lifesciences.cornell.edu/brcinfo/).

Eight primer pairs were used to PCR amplify autosomal microsatellite loci (*Oz06*, *Oz08*, *Oz22*, *Oz27*, *Oz30*, *Oz41*, *Oz43*, *Oz44*; [19]) from a total of 85 individuals: 34 from AP, 33 from ME, and 18 from NH. Multiplexed reactions were carried out in 10 µL volumes containing approximately 1 µL template DNA, 5 µLType-It 2× master mix, 1 µL of multiplexed primer mix (containing 0.2 µM of each primer) and 3 µL RNAse-free water. Reaction conditions included an initial denaturation of 95°C for 5 minutes, 28 cycles of 95°C for 30 seconds, 60°C for 1 minute, 72°C for 30 seconds, and a final elongation at 60°C for 30 minutes. Amplified products were verified on a 2% agarose gel. Successful amplifications were prepared for analysis with formamide and LIZ ladder, and were run on an Applied Biosystems 3730 DNA Analyzer at the Life Sciences Core Laboratories Center at Cornell University (http://cores.lifesciences.cornell.edu/brcinfo/).

### Mitochondrial DNA Analyses

Amplification resulted in 872 base pairs of cytochrome *b* sequence. Sequences were assembled and aligned using CodonCode Aligner v.3.7.1 (CodonCode Corporation, Dedham, MA). Tajima's D [20], Fu's F [21], and measures of genetic diversity (haplotype number, number of segregating sites, nucleotide diversity (π), haplotype diversity) were calculated using DnaSP v.5 [22]. DnaSP was also used to produce mismatch distributions, which display the frequency and distribution of pairwise genetic differences between individuals [23]. The observed data were compared to expected distributions for populations at demographic equilibrium and expansion. Harpending's raggedness statistic, *r*, was calculated to determine the goodness of fit of the data for each expected distribution [23]. A population in demographic equilibrium is expected to have a ragged L-shaped distribution reflecting the stochastic shape of gene trees.

jModelTest [24] was used to determine the appropriate model of evolution and associated parameters for phylogenetic analysis. A neighbor-joining tree using jModelTest's suggested HKY85 [25]+I parameters, bootstrapped for 1,000 iterations, was created in PAUP* v.4 [26]. TCS v.1.21 [27] was used to create a minimum spanning haplotype network through statistical parsimony analysis, where the combined probability of joining the most similar haplotypes is >95%.

### Microsatellite DNA Analyses

Microsatellite alleles were sized and analyzed using GENEMAPPER v.4 (Applied Biosystems). MICRO-CHECKER v.2.2.3 [28] was used to detect the presence of null alleles, allelic dropout and stuttering with a 95% confidence interval. Locus *Oz22* was found to have a high frequency of null alleles and was removed from further analysis. Allele frequencies and number, pairwise differentiation (F_ST_), and observed (H_o_) and expected (H_e_) heterozygosity were calculated using MSA v.4.05 [29]. GENEPOP v.4.1.3 [30] was used to detect deviations from Hardy-Weinberg equilibrium and the presence of linkage disequilibrium, and to calculate pairwise Rho_ST_. The measure Rho_ST_ is similar to F_ST_, but takes allele size into account and has been suggested to be a more accurate estimate of population differentiation under microsatellite evolution conditions [31].

Population structure was assessed using the Bayesian clustering method STRUCTURE v.2.3 [32]. The program was first run for each putative subpopulation number (K), for K = 1–10 in 10 independent runs with an initial burn-in period of 5,000 followed by 50,000 Markov Chain Monte Carlo (MCMC) iterations under an admixture model. The most probable K was approximated by comparing the likelihood (LnP(D)) with different second derivative values of K and selecting the largest ΔK value, following the Evanno method [33]. This resulted in K = 2 as the most probable K. STRUCTURE was then re-run independently with K = 2 and K = 3 for 10 independent runs with an initial burn-in period of 50,000 followed by 500,000 MCMC iterations under a model of correlated allele frequencies. The output from these simulations was then summarized by STRUCTURE HARVESTER online [34] and CLUMPP [35], and graphically displayed using DISTRUCT [36].

We implemented two approaches in evaluating past bottlenecks, as commonly used in conservation genetic studies: BOTTLENECK and *M*-ratio. The software BOTTLENECK v.1.2.02 [37] was used to assess possible genetic bottlenecks by assuming that excess H_e_ relative to H_eq_ (heterozygosity relative to that expected at equilibrium, given the number of alleles), is a signal of a recent decrease in effective population size. One thousand independent simulations were run assuming an infinite alleles model (IAM) [38] and a step-wise mutation model (SMM) [39]. We also ran these simulations using a two-phase mutation model (TPM) [40], which is thought to be intermediate between SMM and IAM, and implements variable proportions of SMM. We tested three different proportions of SMM using this TPM model: 10, 50, and 70%. Piry et al. [37] recognize that IAM and SMM represent two extremes and that the true model of mutation for most loci likely lies in-between them, although it has been suggested that SMM is a more appropriate model for microsatellite evolution [41]. We performed a Wilcoxon signed-rank test [42], a sign test, and an analysis of allele frequency mode shifts [43].

The *M-*ratio method as implemented in M P VAL [44] was used as an alternative method of assessing the probability of a historic bottleneck. The *M*-ratio is defined as M = k/r, the mean ratio of the number of alleles (k) to the size range of those alleles (r) per locus, for a sample of microsatellite loci. The significance of this ratio was determined by comparing the observed *M*-ratio to a simulated population with the same mutational model and sample size in the program CRITICAL M [44]. This program generates a critical value, M_c_, which was set at the lower 5% tail of the distribution. An observed *M*-ratio that falls below this M_c_ constitutes strong evidence that a bottleneck has occurred.

The input of three TPM (two-phase mutation model) parameters is necessary for the calculation of M_c_: *p_s_* (frequency of single step mutations), Δ_g_ (the size of non one-step changes) and θ ( = 4N_e_μ). The value of *p_s_* = 0.88 was taken from [44]. Because no species-specific values exist for muskrats for these parameters and M_c_ can be sensitive to Δ_g_ and Θ, values for Δ_g_ were varied between 2.8 (value obtained from a review by [44]) and 3.5 (default value) [44]. A range of pre-bottleneck θ values were estimated using two long-term estimator equations (e.g., as done in [45]), given the microsatellite heterozygosity values for the mainland and island and μ = 5.0×10^−4^ mutations/locus/generation [46].
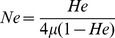
(1)

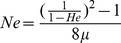
(2)
Equation 1 assumes IAM [47] and Equation 2 assumes SMM [39], thus providing estimates for the two extremes of the mutation process. For independent comparison, we generated maximum likelihood estimates of θ using the program MIGRATE [48].

## Results

### Mitochondrial DNA

Twelve polymorphic nucleotide sites were detected in 872 bp of cytochrome *b* sequence in the 79 individuals analyzed, resulting in 11 unique cytochrome *b* haplotypes, A–K (Table 1). The AP population consistently exhibited lower levels of genetic diversity as compared with the mainland populations of ME and NH (Table 2). Neither Tajima's D nor Fu's F was significantly positive or negative, although AP exhibited strongly positive values (Table 2). A positive Tajima's D signifies a recent bottleneck or balancing selection; similarly, a positive Fu's F_s_ indicates a deficiency of alleles from a recent bottleneck.

**Table 1:** Mitochondrial cytochrome *b* haplotype identities.

Haplotype	Genbank #	Position in Sequence (1–872 bp)											
		186	221	271	450	545	566	568	569	571	618	731	851
A (LLT003)	JX435805	G	T	T	T	C	A	T	A	T	G	T	C
B (BAT003)	JX435799	.	.	.	.	.	.	.	.	.	A	C	.
C (ADV001)	JX435800	.	.	.	.	.	.	.	.	.	.	C	.
D (ADV015)	JX435801	.	C	.	C	.	.	.	.	.	.	.	.
E (RWC004)	JX435802	.	C	.	.	.	.	.	.	C	.	.	.
F (ADV004)	JX435803	.	C	.	.	.	.	.	.	.	.	.	.
G (WA001)	JX435804	.	C	C	.	.	.	.	G	.	.	.	.
H (BAT001)	JX435798	.	.	.	.	.	.	C	.	.	.	C	.
I (WA006)	JX435806	.	.	.	.	.	G	.	.	.	.	.	.
J (AMM001)	JX435807	A	.	.	.	A	.	.	.	.	.	.	T
K (RWC001)	JX435808	A	.	.	.	.	.	.	.	.	.	C	T

Haplotypes are referred to by alphabetical letter A–K; a representative sequence of each haplotype, denoted by sample ID in parentheses, has been deposited in GenBank. See Table S1 for additional specimen information.

**Table 2:** Measures of genetic diversity for 872 bp of cytochrome *b*.

Population	N	H	S	π	H_d_	Tajima's D*	Fu's F_s_ *
AP	33	2	4	0.00174	0.379	1.345	5.007
ME	31	8	8	0.00209	0.789	−0.269	−1.545
NH	15	5	7	0.00179	0.562	−0.988	−0.416
Mainland	46	11	12	0.00211	0.754	−0.982	−3.417

The mainland represents ME and NH pooled. N = number of sequences. H = number of haplotypes. S = number of segregating sites. π = nucleotide diversity. H_d_ = haplotype diversity.

*For neutrality statistics, Tajima's D and Fu's F_s_, *P*>0.10.

**Figure 2:**
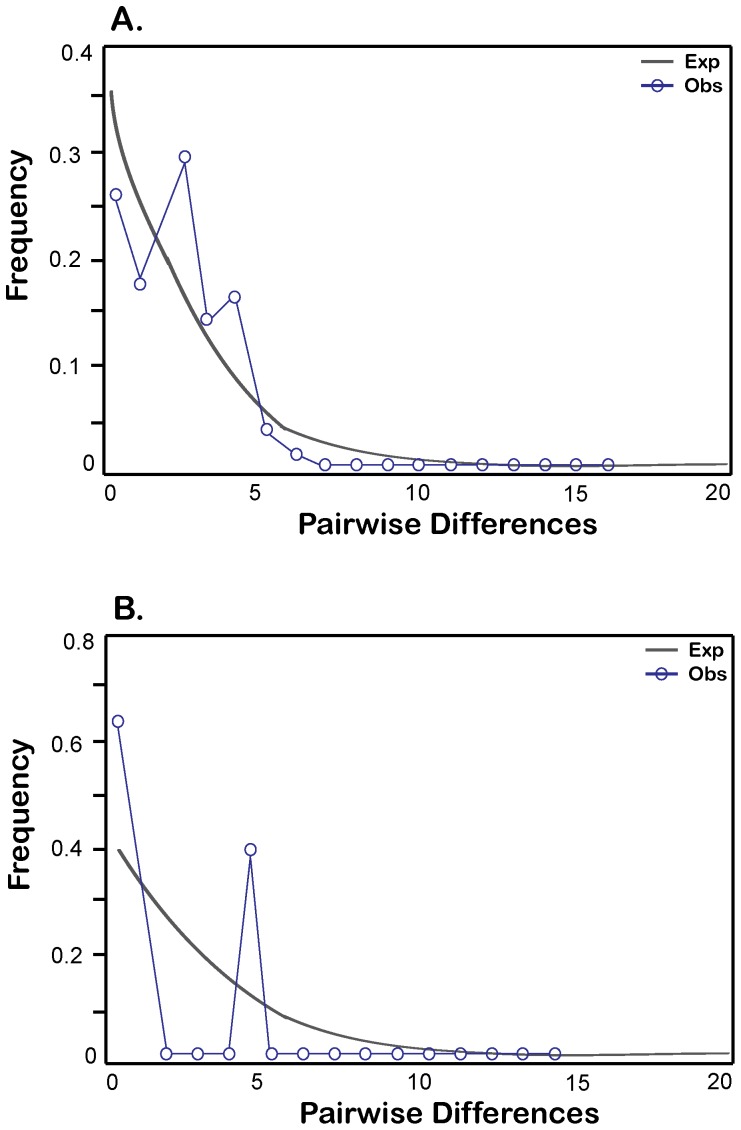
Mismatch distribution for A. the mainland population, ME and NH pooled, and B. AP only. Frequency is represented on the vertical axis. Solid gray lines indicate expected values and blue circles represent observed values. The expected frequency is based on a constant population size and demographic equilibrium. Raggedness statistics, *r* = 0.0592 and 0.6729 for **A**. and **B**., respectively.

Mismatch distributions plotted for mainland sequences (ME and NH were pooled, as microsatellite analyses revealed little population subdivision) corresponded to a model for expected values of demographic stability, as suggested by the small value of *r* = 0.06 (Figure 2A). Conversely, for the AP sample, a value of *r* = 0.67 and a bimodal distribution suggested a poor fit to a model of demographic stability (Figure 2B).

**Figure 3:**
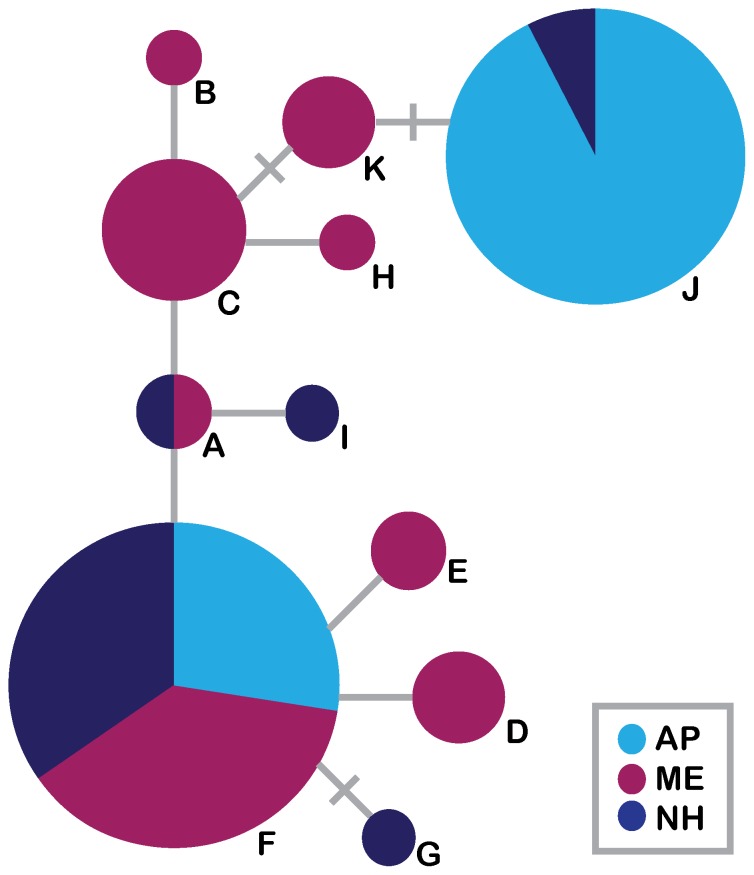
TCS haplotype network for mitochondrial cytochrome *b* haplotypes. Size of circles is proportional to the number of individuals with that haplotype, where the largest circle depicts 25+ individuals and the smallest depicts one individual. Letters correspond to haplotypes described in Table 1. Each node represents a 1-bp change in nucleotide sequence, and hashes along a node represent probable missing haplotypes. Light blue shows AP, dark blue shows NH, and dark pink shows ME. Note that haplotype J is only found in AP and the coastal port of Portsmouth, NH.

The TCS statistical parsimony network revealed relationships among haplotypes identical to those portrayed by a neighbor-joining tree (Figure 3; Figure S2). Despite multiple analyses revealing little differentiation between ME and NH and the fact that political boundaries are frequently not biologically meaningful, we retain these states as population units when showing levels of genetic variation, in addition to a combined “mainland”, to display phylogeographic relationships within the mainland, facilitate data sharing with state wildlife agencies, and allow for comparison with data from previous studies of Canada and other US states [11]. Two haplotypes (F and J) occur at high frequency in our total sample and represent the two haplotypes present on AP. F is the most common and widely distributed haplotype, with individuals from ME, AP, and NH. J was found in the majority of AP muskrats and in NH muskrats from the coastline. No haplotypes were endemic to AP. Phylogenetic clustering of haplotypes by geographic origin was not evident, but the frequency of a given haplotype varied in geographic distribution between ME and NH. Haplotypes F and J show no evidence of geographic structure within AP (Figure S1).

### Microsatellite DNA

Null alleles and inconsistent peak morphology were present in one locus, *Oz22*, and this locus was not included in further analyses. Ninety-four alleles were detected at 7 microsatellite loci for 85 muskrats. No significant deviation from Hardy-Weinberg was found (P >0.05 for each locus across each population), and there was likewise no evidence of linkage disequilibrium (P >0.05 for each locus pair across all populations). The AP population exhibited the fewest number of alleles per locus, lowest H_e_ and H_o_, and lacked private alleles (Table 3). A Fisher's exact G-test revealed that allele frequencies per locus and across all loci are significantly different between AP as compared with ME and NH (*P*<0.0001). Little pairwise differentiation between ME and NH was found, with F_ST_ = 0.028 (Table 4). Conversely, high levels of differentiation were detected in pairwise comparisons of ME and NH with AP (F_ST_ = 0.149–0.166; P<0.0001) (Table 4). Values of pairwise Rho_ST_ (Table S2), displayed a pattern consistent with all values of F_ST_.

**Table 3:** Summary of microsatellite variation for 85 muskrats (34 AP, 33 ME, 18 NH) at 7 loci.

	AP			ME			NH			Mainland		
	H_o_	H_e_	A	H_o_	H_e_	A	H_o_	H_e_	A	H_o_	H_e_	A
*Oz06*	0.50	0.57	3	0.85	0.88	12	0.78	0.86	10	0.70	0.83	14
*Oz08*	0.53	0.60	4	0.79	0.86	8	0.72	0.83	7	0.68	0.80	9
*Oz27*	0.70	0.68	4	0.88	0.86	8	0.72	0.83	7	0.78	0.83	9
*Oz 30*	0.68	0.73	7	0.85	0.89	15	0.83	0.91	12	0.78	0.88	18
*Oz41*	0.59	0.63	6	0.97	0.93	21	0.83	0.88	11	0.79	0.89	23
*Oz43*	0.41	0.45	3	0.73	0.83	12	0.83	0.87	13	0.63	0.82	16
*Oz44*	0.38	0.44	3	0.24	0.29	4	0.44	0.46	4	0.34	0.40	5
Mean	0.54	0.59	4.3	0.76	0.79	9.4	0.74	0.80	9.1	0.67	0.78	13.4

H_o_ and H_e_ are observed and expected heterozygosity. A = number of alleles per locus. Bottom row represents values averaged across all loci.

**Table 4:** F_ST_ values calculated for microsatellite DNA at seven loci, *P*<0.0001 for all pairwise comparisons.

	AP	ME	NH	Mainland
AP	—	0.166	0.155	0.149
ME	0.166	—	0.028	—
NH	0.155	0.028	—	—
Mainland	0.149	—	—	—

Mainland is ME and NH combined as a single population.

**Figure 4:**
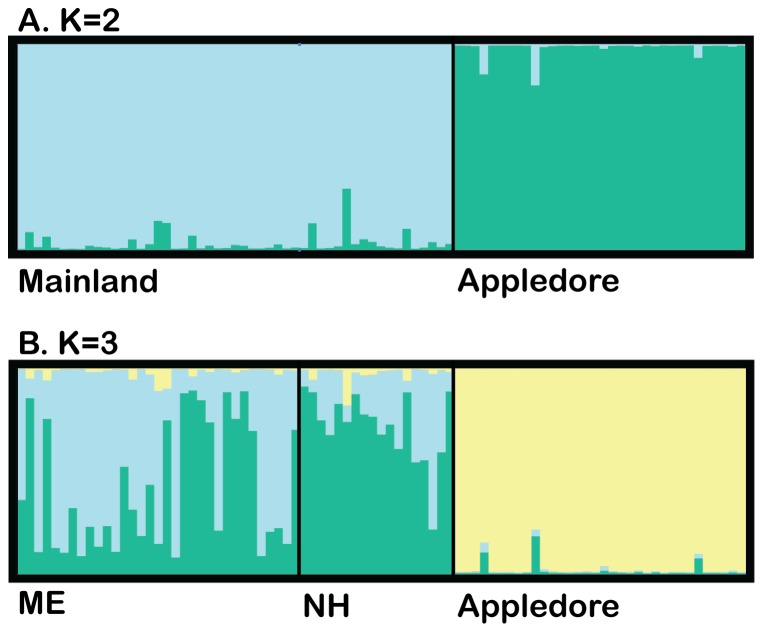
Assignment of individuals to clusters using STRUCTURE and displayed with DISTRUCT, with A. K = 2 and B. K = 3. K = 2 is the true value of K (Figure S3), with K = 3 provided for comparison. Individuals are grouped by region sampled, with K = 2 including ME and NH as a single “mainland” cluster. Y axis represents percentage of ancestry in each cluster.

Applying the Evanno method [33], the most probable number of population clusters in STRUCTURE was determined to be K = 2 (Figure S3). These two clusters corresponded to AP as a cluster separate from the mainland regions of ME and NH pooled (Figure 4A). A hypothesis testing ME and NH as distinct clusters with K = 3 revealed very little evidence of differentiation across the mainland (Figure 4B). AP was found to be a distinct cluster in both K = 2 and K = 3.

Analysis of potential bottlenecks using BOTTLENECK produced equivocal outcomes, with results differing between use of the infinite alleles model (IAM) [38], step-wise mutation model (SMM) [39], and two-phase mutation model (TPM) [40]. Both a sign and Wilcoxon test rejected a neutral model for heterozygosity excess for simulations under IAM, but did not for SMM or TPM (Table S3). No mode shift was detected, and there was a normal L-shaped distribution of allele frequencies.

*M-*ratio analyses and simulations produced significant evidence for a historical bottleneck. Estimations of N_e_ for the current island population drawn from Equations 1 and 2 ranged from 720–1237 individuals, a value within the upper bound of estimates from past mark-recapture studies [17]. MIGRATE generated θ values within this range (1.4–1.7) for microsatellite data. All observed *M*-ratio values were below the critical simulated value, M_c_, when Δ_g_ = 2.8 (Table 5). Consistent with the fact that increasing values of Δ_g_ are known to strongly erode the bottleneck signature [44], fewer *M*-ratios fell below the simulated M_c_ when Δ_g_ = 3.5; however, the majority were still smaller than M_c_, and all were smaller than the average simulated *M*-ratio, M_avg_.

**Table 5:** Summary of parameters used in *M*-ratio analyses used to detect significant reductions in population size.

Ne	Θ	p_s_	*M*-ratio	M_avg_ a	M_c_ a	M_avg_ b	M_c_ b
10000	20	0.88	0.4172	0.604	0.5137	0.5183	0.4356
5000*	10	0.88	0.4172	0.6206	0.5257	0.5318	0.444
2000*	4	0.88	0.4172	0.6189	0.5203	0.5281	0.4335
1000*	2	0.88	0.4172	0.6053	0.5002	0.5178	0.4171
750*	1.5	0.88	0.4172	0.6009	0.4972	0.515	0.4014
500	1	0.88	0.4172	0.5958	0.4901	0.5169	0.4068
100	0.2	0.88	0.4172	0.6065	0.4952	0.5481	0.4226
50	0.1	0.88	0.4172	0.6113	0.5	0.5545	0.4265
10	0.02	0.88	0.4172	0.6162	0.5071	0.5641	0.4337

M_avg_ is average simulated *M*-ratio value for a given set of parameters and empirical microsatellite data; M_c_ is the critical value. M_avg_ and M_c_ were generated for Δ_g_ values of 2.8 and 3.5.

* indicates N_e_ values within the range calculated with long-term estimators.

a = **Δ_g_** value of 2.8;

b = **Δ_g_** value of 3.5.

## Discussion

### Multiple Lines of Evidence

The genetic data reported here are congruent with anecdotes suggesting that humans introduced muskrats in the early 1900s for the purposes of fur harvest. Although on their own, these data are not sufficient to unequivocally warrant an “introduced” status, several other lines of independent evidence support such a designation. Morphologically, AP muskrats have slightly narrower crania than mainland muskrats [17], but overall size and morphological variability are very similar, suggesting that this population has not evolved in isolation over a long period of time. Muskrats do not appear in the zooarchaeological record within the Isles of Shoals until approximately 80–100 years ago (N. Hamilton *personal communication*), despite the presence of human artifacts and even Norway rat bones several hundred years earlier [8]–[9]. Interviews conducted in the 1980s with local fishermen and trappers suggest that muskrats were already present on AP in the 1930s [17].

Comparison with other insular mammal populations of land bridge islands in the northern Gulf of ME also supports a human-mediated introduction. From a source pool of ∼33 species of mammals native to the state of ME, only muskrats are present on AP. Crowell [49] found that island area and isolation explain the most variation in species richness for coastal islands in the northern Gulf of ME, where there are indeed native muskrat populations. However, those islands are orders of magnitude larger than AP and are also far closer to a mainland source pool. For example, the island Gran Manan is a similar distance from a mainland area (10 km), but is several hundred times larger than AP, and harbors relictual populations of only 4 mammalian species, including voles, mice, and chipmunks; it does not harbor muskrats. Within coastal islands closer to the source pool, the smallest islands marginally comparable to AP only have voles; overall, we see distinctive, nonrandom patterns of species composition, where small islands contain a nested subset of mammal species found on larger islands [49]. This implies that if mammals have colonized AP naturally via land bridge, we would see the smallest subset, such as voles and mice; instead, we see only muskrats, suggesting a muskrat-specific process.

Crowell [49] concludes his comprehensive study of 24 land bridge coastal islands by suggesting that that there is strong evidence that most mammals are able to cross salt-water barriers 0.5–1.5 km wide. Given the swimming ability of semiaquatic rodents such as beavers and muskrats, it is no surprise that they can cross even wider water barriers of up to a maximum of 5 km [49]. However, AP is 11 km from a mainland source pool, thus arguing against sustained migration or a natural dispersal event and consistent with the high levels of genetic differentiation between the mainland and AP. Biogeographic expectations are consistent with our genetic data, as AP lacks any private alleles or unique haplotypes suggesting that the population has not had sufficient time to generate endemic insular variants.

### Genetic Diversity

Founder events involve a subsample of the individuals from a source population, resulting in decreased genetic variation and increased divergence between source and founder populations [50]. Both cytochrome *b* sequences and microsatellite data of AP muskrats revealed consistently lower levels of genetic diversity as compared to the mainland source. Both ME and NH exhibited levels of heterozygosity comparable to those found for muskrats inhabiting nearby regions of NY, Quebec and New Brunswick, with H_o_ = 0.68–0.83 [11]; the average H_o_ was 0.54 for the AP muskrats.

### Population Differentiation

As evidenced from the lack of phylogeographic grouping within the haplotype genealogies and the STRUCTURE results, muskrats in ME and NH show no evidence of geographic structure, presumably reflecting ongoing gene flow. The Bayesian clustering analysis performed using STRUCTURE suggests that AP is a cluster distinct from the mainland regions, discrediting the hypothesis of recent gene flow that would result from continued migration by swimming. This was confirmed by significant, elevated pairwise F_ST_ values.

### Evidence of a Bottleneck

Both mitochondrial and microsatellite DNA markers were used to assess evidence for a recent bottleneck associated with a human-mediated colonization of AP. Using mitochondrial markers, we found that Fu's F_s_ [21] and Tajima's D [20] for AP sequences were not significantly different from zero. However, both exhibited large positive values, which would suggest a demographic contraction, particularly due to a bottleneck [51]. Other studies of historic bottlenecks have found similar positive, yet non-significant values. For example, Weber et al [52] calculated F_s_ and D values for a fur seal population that underwent a known bottleneck in the 19^th^ century due to overhunting. They reported values for F_s_ of 4.423 and D of 1.35, although neither was significant. These values, derived from a species with a known historic bottleneck, are similar to those reported here for muskrats: F_s_ = 5.007, D = 1.35. The mismatch distribution for AP resulted in a rejection of a constant population size, as interpreted from a high raggedness statistic [23] and a bimodal distribution; Weber et al [52] also report a bimodal distribution in fur seals.

The program BOTTLENECK produced ambiguous results. During a founder event, rare alleles are more likely to be lost than common variants [47]. This allows for bottleneck detection by two methods: identifying a temporary excess of heterozygosity (relative to that expected, given the number of alleles) and a departure from an L-shaped allele frequency distribution [43]. Simulations run assuming IAM indicated the presence of a bottleneck, but more conservative simulations using SMM and TPM were not significant. No departure from an L-shaped allele frequency distribution was detected.

The power of BOTTLENECK's analyses is largely dependent on the generation time of the focal species and the size of the effective founding population, N_e_. Heterozygosity excess is a transient signature only detectable for 0.2–4.0(N_e_) generations after a bottleneck event [53]. Detection of heterozygosity excess may be hindered due to population-specific processes, such as explosive population growth following an initial colonization event, which would diminish the loss of rare alleles and prevent drastic reduction of H_eq_. Indeed, populations with an explosive growth rate can still experience overall reductions in genetic diversity but not display heterozygosity excess due to a bottleneck [50]. If a study evaluating a potentially introduced species, such as this one, employed only this technique, it could erroneously suggest the absence of a bottleneck, obscuring the detection of a cryptic introduction that occurred in historic (within the last few hundred years) but not recent times [2].

A combined approach that includes analyzing *M*-ratios, allele frequency shifts, and heterozygosity excess can provide windows into population decline and recovery across different timescales and thus provide a method of comparison for hypotheses of bottleneck timing. Heterozygosity excess and allele frequency distributions recover faster than the *M*-ratio [44]. For a population that has experienced historic declines, such as the bottleneck in the early 1900s for muskrats, we would expect to see a low, significant *M*-ratio juxtaposed with non-significant heterozygosity excess and allele frequency shifts— this is exactly the result reported here. Conversely, a recent bottleneck, such as if muskrats were introduced very recently in 1990, would show significant signatures in both slowly and rapidly recovering measures [54].

### Source Population

Mitochondrial DNA revealed 11 distinct haplotypes found in 79 muskrats, and only 2, F and J, on AP. From haplotypes alone, it is possible that the AP muskrats are either (1) solely from the NH coastline, where both haplotypes F and J co-occur; or (2) from both ME and NH, with the island's F lineage coming from ME (given the high frequency of the F haplotype in ME). The coastal area of NH is the only region sampled that contains the 2 haplotypes also present on AP, making it the most probable source population (however, we stress such haplotype data are suggestive but not conclusive). In addition, this coastal area is a major shipping, fishing, and naval shipyard (Portsmouth, NH), with high levels of boat traffic to the Isles of Shoals. Although we recognize that the distinction between ME and NH is politically and economically, rather than biologically, meaningful, retaining such a distinction between state-lines with different human dynamics (such as differing shipping routes and local economies) allows us to glean further insight into the human dimension of the muskrat colonization event.

An intentional introduction in the early 1900s is consistent with the genetic data presented here. In her observations on the Isles of Shoals' natural history, Celia Thaxter presciently contemplated “how the [Norway] rats came here first is not known; probably some old ship imported them” [55]. The accidental and intentional translocation of numerous species has resulted in novel island communities, often to the detriment of endemic species, and may go undetected due to slight morphological or ecological divergence upon colonization. While there are numerous reasons to exercise caution when reconstructing the past trajectories of introduced species using limited genetic data [56], genetic data when contextualized appropriately, as in this case, can be seen as one of many lines of independent evidence supporting an introduced status. Although muskrats in wetland areas are often destructive to human industry, the muskrats introduced to AP do not appear to have any urgent negative impacts on human activities or native fauna, including the populations of colonial ground-nesting birds; therefore, no eradication actions are currently proposed. However, whether detrimental effects do arise from a growing muskrat population (particularly due to a lack of natural predators), decisions regarding muskrat removal will not be impeded by the question of if the AP muskrats are an endemic island subspecies in need of protection, as based on our study it is clear that they are instead non-native visitors to the Isles of Shoals.

## Supporting Information

Figure S1 **Map of Appledore Island and muskrat sampling locations.** Red circles are haplotype J, blue circles are haplotype F, and purple circles are microsatellite data only. Sampling was conducted in thoroughly surveyed muskrat habitat; areas of the island that did not yield samples were occupied by highly aggressive nesting seabirds (which are a significant deterrent to muskrats), lacked appropriate vegetation, or were not in close proximity to fresh water. The results of our habitat use surveys were congruent with those of a 1984 study [17]. Basemap sources: ESRI, USGS.
(TIF)

Figure S2 Neighbor-joining tree of all haplotypes (872 base pairs of cytochrome b) using HKY85+I parameters, made in PAUP* v.4. Letters correspond to haplotypes, with specimen IDs in parentheses. Geographic information for each specimen can be found in Table S1.
(TIF)

Figure S3 **ΔK plot for STRUCTURE data.** The value of ΔK peaks strongly at K = 2 (341.2816), declines abruptly to K = 3 (3.6114), and continues to decline for values K = 4 and greater.
(TIF)

Table S1 **List of all specimens used in this study, their mitochondrial haplotype, and their geographic coordinates.** * indicates specimen only yielded microsatellite data. See Table 1 for Genbank IDs for each haplotype.
(DOCX)

Table S2 **Results of pairwise Rho_ST_ for microsatellite DNA at seven loci.** Mainland is ME and NH combined as a single population.
(DOCX)

Table S3 **Results of sign and Wilcoxon sign-rank tests from BOTTLENECK simulations under an infinite alleles model (IAM), step-wise mutation model (SMM), and two-phase mutation model (TPM).** A significant P value indicates excess heterozygosity, indicative of a bottleneck. *For TPM, we report only 10% SMM values as illustrative of our TPM results. All results for TPM regardless of proportion of SMM in the model (10%, 50%, 70% SMM) were P>0.05 and non-significant. a = P<0.05, b = P<0.01, c = P<0.005.
(DOCX)
