# Supplementary figures and images for: Genetics Reveal the Origin and Timing of a Cryptic Insular Introduction of Muskrats in North America
Source: PLoS One. 2014 Oct 31;9(10):e111856. doi: 10.1371/journal.pone.0111856 (PMC4216123; doi:10.1371/journal.pone.0111856)

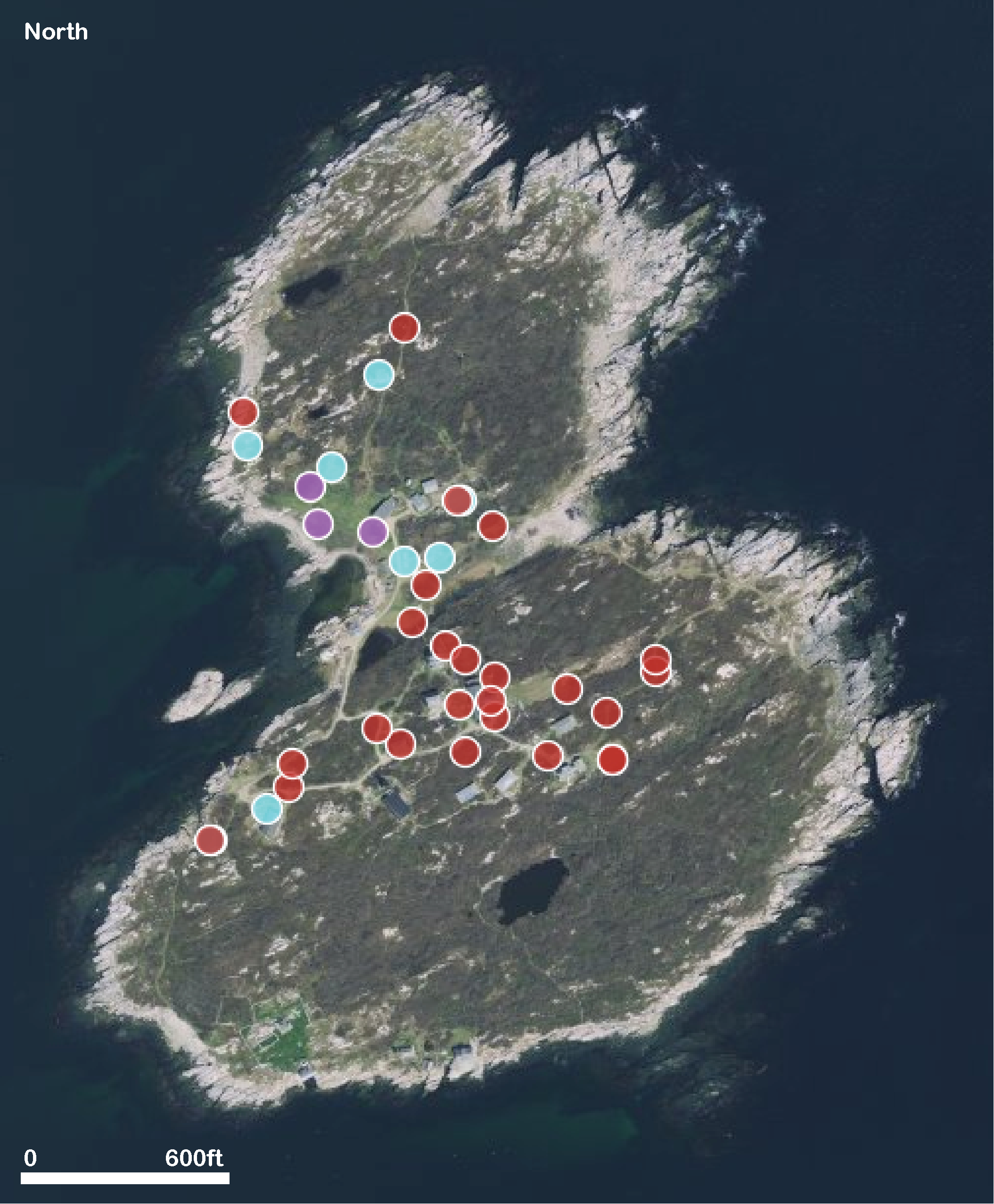

Supplement: Figure S1 — Map of Appledore Island and muskrat sampling locations. Red circles are haplotype J, blue circles are haplotype F, and purple circles are microsatellite data only. Sampling was conducted in thoroughly surveyed muskrat habitat; areas of the island that did not yield samples were occupied by highly aggressive nesting seabirds (which are a significant deterrent to muskrats), lacked appropriate vegetation, or were not in close proximity to fresh water. The results of our habitat use surveys were congruent with those of a 1984 study [17]. Basemap sources: ESRI, USGS. (TIF) [file pone.0111856.s001.tif]

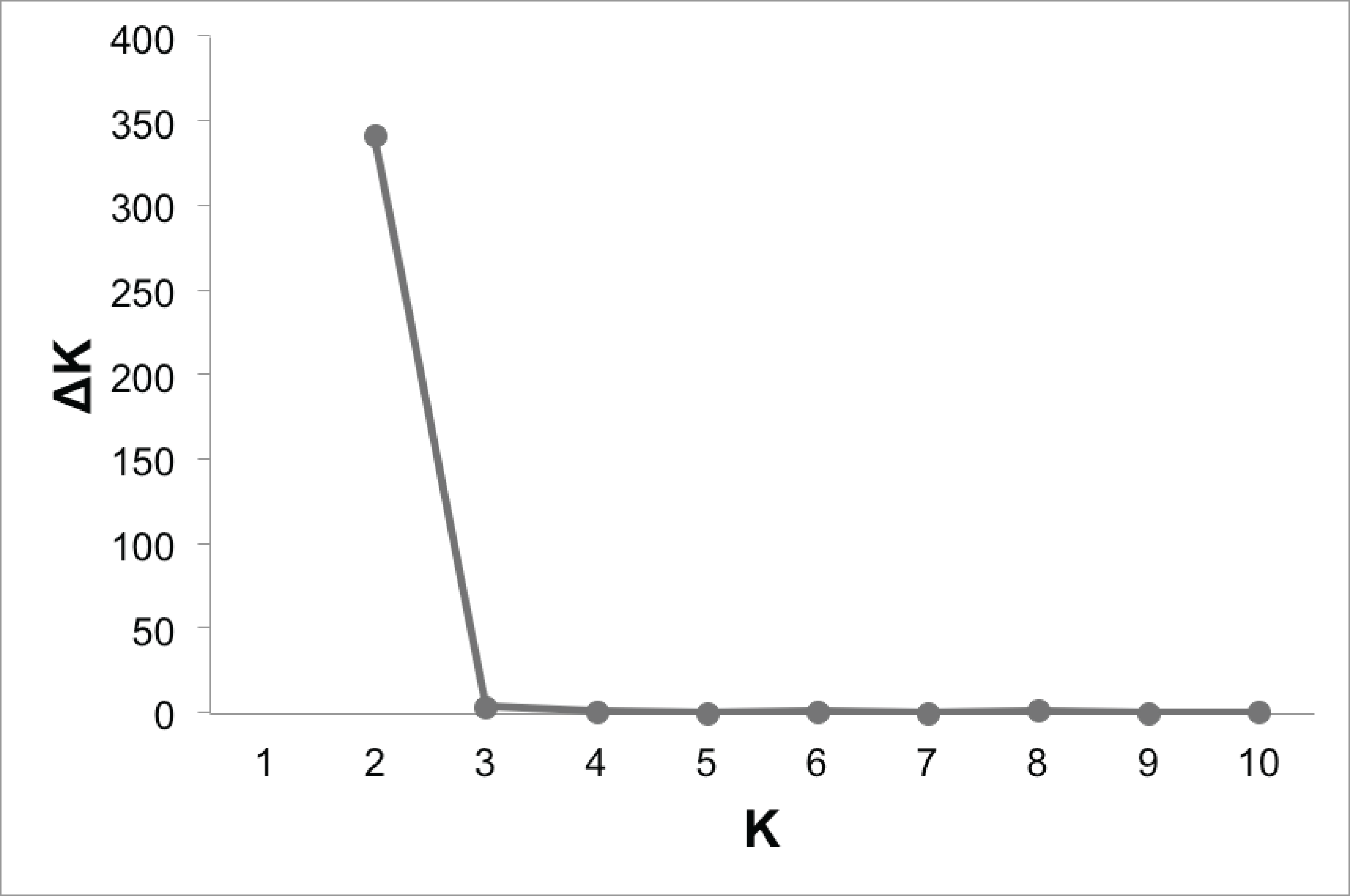

Supplement: Figure S3 — ΔK plot for STRUCTURE data. The value of ΔK peaks strongly at K = 2 (341.2816), declines abruptly to K = 3 (3.6114), and continues to decline for values K = 4 and greater. (TIF) [file pone.0111856.s003.tif]
