# Supplementary material for: Genetics Reveal the Origin and Timing of a Cryptic Insular Introduction of Muskrats in North America
Source: PLoS One. 2014 Oct 31;9(10):e111856. doi: 10.1371/journal.pone.0111856 (PMC4216123; doi:10.1371/journal.pone.0111856)
Supplement: Table S1 — List of all specimens used in this study, their mitochondrial haplotype, and their geographic coordinates. * indicates specimen only yielded microsatellite data. See Table 1 for Genbank IDs for each haplotype. (DOCX) [file pone.0111856.s004.docx]

| Sample ID | Sampling Area | Haplotype | Latitude | Longitude |
| --- | --- | --- | --- | --- |
| AMM005* | AP | — | 42°59'21.15"N | 70°36'57.96"W |
| AMM034* | AP | — | 42°59'22.24"N | 70°36'58.25"W |
| AMM037* | AP | — | 42°59'20.90"N | 70°36'55.76"W |
| AMM006 | AP | F | 42°59'12.95"N | 70°36'59.94"W |
| AMM010 | AP | F | 42°59'25.44"N | 70°36'55.55"W |
| AMM019 | AP | F | 42°59'20.08"N | 70°36'54.50"W |
| AMM022 | AP | F | 42°59'22.81"N | 70°36'57.35"W |
| AMM031 | AP | F | 42°59'12.08"N | 70°37'2.10"W |
| AMM033 | AP | F | 42°59'21.80"N | 70°36'52.31"W |
| AMM035 | AP | F | 42°59'20.18"N | 70°36'53.14"W |
| AMM041 | AP | F | 42°59'23.42"N | 70°37'0.73"W |
| AMM001 | AP | J | 42°59'14.61"N | 70°36'52.14"W |
| AMM002 | AP | J | 42°59'15.62"N | 70°36'50.94"W |
| AMM003 | AP | J | 42°59'15.93"N | 70°36'52.38"W |
| AMM004 | AP | J | 42°59'19.41"N | 70°36'53.71"W |
| AMM007 | AP | J | 42°59'14.50"N | 70°36'48.92"W |
| AMM008 | AP | J | 42°59'16.76"N | 70°36'50.98"W |
| AMM009 | AP | J | 42°59'17.66"N | 70°36'52.88"W |
| AMM011 | AP | J | 42°59'24.36"N | 70°37'0.88"W |
| AMM012 | AP | J | 42°59'12.08"N | 70°37'2.17"W |
| AMM013 | AP | J | 42°59'14.82"N | 70°36'54.65"W |
| AMM014 | AP | J | 42°59'18.31"N | 70°36'54.25"W |
| AMM015 | AP | J | 42°59'14.39"N | 70°36'46.33"W |
| AMM016 | AP | J | 42°59'17.16"N | 70°36'47.48"W |
| AMM017 | AP | J | 42°59'16.40"N | 70°36'48.10"W |
| AMM018 | AP | J | 42°59'13.60"N | 70°36'59.08"W |
| AMM021 | AP | J | 42°59'26.81"N | 70°36'54.54"W |
| AMM023 | AP | J | 42°59'15.29"N | 70°36'55.58"W |
| AMM024 | AP | J | 42°59'17.23"N | 70°36'52.16"W |
| AMM025 | AP | J | 42°59'15.72"N | 70°36'46.58"W |
| AMM027 | AP | J | 42°59'21.84"N | 70°36'52.42"W |
| AMM028 | AP | J | 42°59'14.24"N | 70°36'58.93"W |
| AMM029 | AP | J | 42°59'16.91"N | 70°36'44.64"W |
| AMM030 | AP | J | 42°59'17.23"N | 70°36'44.64"W |
| AMM032 | AP | J | 42°59'21.12"N | 70°36'51.05"W |
| AMM042 | AP | J | 42°59'16.09"N | 70°36'51.16"W |
| ADV019* | ME | — | 44°53'15.36"N | 69°16'31.44"W |
| ADV022* | ME | — | 44°53'28.71"N | 69°16'7.35"W |
| ADV023* | ME | — | 45°13'3.36"N | 69°58'39.72"W |
| MAC003* | ME | — | 44°19'33.09"N | 69°45'54.51"W |
| ADV014 | ME | A | 43°48'39.24"N | 70°40'34.68"W |
| BAT003 | ME | B | 44°12'25.37"N | 69°48'13.22"W |
| ADV001 | ME | C | 44°53'15.00"N | 69°16'31.08"W |
| ADV002 | ME | C | 44°53'22.56"N | 69°16'39.00"W |
| ADV006 | ME | C | 44°53'15.00"N | 69°16'31.10"W |
| ADV009 | ME | C | 44°51'19.80"N | 69°16'17.04"W |
| ADV011 | ME | C | 44°50'54.24"N | 69°12'38.88"W |
| ADV020 | ME | C | 45°12'16.20"N | 69°39'59.04"W |
| ADV021 | ME | C | 44°53'28.69"N | 69°16'7.33"W |
| ALP002 | ME | C | 45°27'59.13"N | 69°42'33.33"W |
| ALP004 | ME | C | 44°18'38.00"N | 69°34'50.00"W |
| ADV015 | ME | D | 44°53'28.68"N | 69°16'6.96"W |
| ADV016 | ME | D | 44°48'39.23"N | 69°13'34.67"W |
| ADV018 | ME | D | 44°53'15.00"N | 69°16'31.08"W |
| RWC004 | ME | E | 44°39'20.72"N | 68°56'15.58"W |
| RWC006 | ME | E | 44°39'38.49"N | 68°55'48.94"W |
| ADV003 | ME | F | 43°53'7.80"N | 70°40'4.32"W |
| ADV004 | ME | F | 43°44'35.88"N | 70°40'14.76"W |
| ADV005 | ME | F | 43°53'7.80"N | 70°75'3.60"W |
| ADV007 | ME | F | 44°51'19.80"N | 69°16'16.68"W |
| ADV008 | ME | F | 44°51'19.80"N | 69°16'16.32"W |
| ADV010 | ME | F | 44°53'22.56"N | 69°16'39.00"W |
| ADV012 | ME | F | 44°53'7.44"N | 69°15'3.60"W |
| ADV013 | ME | F | 44°48'1.44"N | 69°12'59.76"W |
| ADV017 | ME | F | 44°51'19.82"N | 69°16'16.68"W |
| BAT002 | ME | F | 44°15'29.00"N | 69°48'29.99"W |
| BAT004 | ME | F | 44°17'42.73"N | 69°46'55.35"W |
| BAT001 | ME | H | 44°12'21.00"N | 69°47'31.00"W |
| RWC001 | ME | K | 44°40'24.45"N | 68°52'55.08"W |
| RWC003 | ME | K | 44°40'31.58"N | 68°53'13.97"W |
| RWC005 | ME | K | 44°40'31.17"N | 68°53'0.03"W |
| WA002* | NH | — | 43°18'40.69"N | 71°10'5.94"W |
| WA003* | NH | — | 43°16'43.06"N | 71° 8'55.81"W |
| WA007* | NH | — | 43°16'21.11"N | 71° 6'28.78"W |
| LLT003 | NH | A | 43° 3'49.66"N | 70°58'14.00"W |
| LLT001 | NH | F | 43° 9'7.95"N | 70°50'9.87"W |
| LLT002 | NH | F | 43° 3'8.76"N | 70°57'4.35"W |
| LLT004 | NH | F | 43° 4'9.89"N | 70°57'8.50"W |
| LLT005 | NH | F | 42°57'46.90"N | 71°21'6.46"W |
| LLT006 | NH | F | 43° 0'8.00"N | 70°57'36.10"W |
| LLT009 | NH | F | 43°10'43.20"N | 70°54'4.70"W |
| WA004 | NH | F | 43°16'43.06"N | 71° 8'55.81"W |
| WA005 | NH | F | 43°16'43.06"N | 71° 8'55.81"W |
| PWD001 | NH | F | 43°51'8.52"N | 71°39'45.50"W |
| PWD002 | NH | F | 43°43'42.50"N | 71°24'51.00"W |
| WA001 | NH | G | 43°15'47.89"N | 71° 8'10.38"W |
| WA006 | NH | I | 43°13'56.70"N | 71°11'11.40"W |
| LLT007 | NH | J | 43° 9'29.40"N | 70°51'47.20"W |
| LLT008 | NH | J | 43°13'57.20"N | 70°50'9.39"W |
