# Supplementary material for: Genetics Reveal the Origin and Timing of a Cryptic Insular Introduction of Muskrats in North America
Source: PLoS One. 2014 Oct 31;9(10):e111856. doi: 10.1371/journal.pone.0111856 (PMC4216123; doi:10.1371/journal.pone.0111856)
Supplement: Table S2 — Results of pairwise RhoST for microsatellite DNA at seven loci. Mainland is ME and NH combined as a single population. (DOCX) [file pone.0111856.s005.docx]

|  | AP | ME | NH | Mainland |
| --- | --- | --- | --- | --- |
| AP | — | 0.2344 | 0.2244 | 0.1995 |
| ME | 0.2344 | — | 0.0774 | — |
| NH | 0.2244 | 0.0774 | — | — |
| Mainland | 0.1995 | — | — | — |
