# Supplementary material for: Genetics Reveal the Origin and Timing of a Cryptic Insular Introduction of Muskrats in North America
Source: PLoS One. 2014 Oct 31;9(10):e111856. doi: 10.1371/journal.pone.0111856 (PMC4216123; doi:10.1371/journal.pone.0111856)
Supplement: Table S3 — Results of sign and Wilcoxon sign-rank tests from BOTTLENECK simulations under an infinite alleles model (IAM), step-wise mutation model (SMM), and two-phase mutation model (TPM). A significant P value indicates excess heterozygosity, indicative of a bottleneck. *For TPM, we report only 10% SMM values as illustrative of our TPM results. All results for TPM regardless of proportion of SMM in the model (10%, 50%, 70% SMM) were P>0.05 and non-significant. a = P<0.05, b = P<0.01, c = P<0.005. (DOCX) [file pone.0111856.s006.docx]

| Sign test | | | Wilcoxon sign-rank one tail | | | Wilcoxon sign-rank two tail | | |
| --- | --- | --- | --- | --- | --- | --- | --- | --- |
| IAM | SMM | TPM* | IAM | SMM | TPM* | IAM | SMM | TPM* |
| 0.0165^a^ | 0.2934 | 0.3623 | 0.0039^c^ | 0.6563 | 0.0547 | 0.0078^b^ | 0.8125 | 0.4832 |
